# Maternal-Neonatal Serum Albumin Level and Neonatal Respiratory Distress Syndrome in Late-Preterm Infants

**DOI:** 10.3389/fped.2021.666934

**Published:** 2021-08-13

**Authors:** Qian Ying, Xue-qin You, Fei Luo, Ji-mei Wang

**Affiliations:** Department of Neonatal, The Obstetrics & Gynecology Hospital of Fudan University, Shanghai, China

**Keywords:** serum albumin, late-preterm infant, maternal-neonatal, respiratory distress syndrome, risk factor

## Abstract

**Background:** To determine the correlation between maternal-neonatal serum albumin level and respiratory distress syndrome (RDS) in late-preterm infants.

**Methods:** This case-control study included 112 late-preterm newborns admitted to the neonatal intensive care unit of our hospital between January 2018 and July 2019. Those infants were divided into the RDS group (*n* = 56) and the non-RDS group (*n* = 56). Levels of maternal-neonatal serum albumin, pregnancy complications, and baseline information of the infants were compared between the two groups.

**Results:** 1. There was no correlation between maternal and neonatal serum albumin measures. The maternal albumin level in the RDS group was lower than that in the control group (33.38 ± 3.31 vs. 33.60 ± 3.31, *P* > 0.05), but the difference was not statistically significant. The neonatal albumin level in the RDS group was significantly lower than that in the control group (32.70 ± 2.48 vs. 35.66 ± 3.27, *P* < 0.05). To predict RDS in late-preterm infants, using the albumin cutoff level of 34 g/L provides a sensitivity of 83.9% with a specificity of 62.5%. 2. Gestational age, primipara, placenta previa, antenatal corticosteroid therapy, delivery mode, and neonatal serum albumin level were associated with RDS in the late-preterm infant. 3. After adjustment for gestational age, logistic regression analysis showed that neonatal serum albumin level, placenta previa, and delivery mode were independent risk factors for RDS in late-preterm infants. However, albumin level did not related to the severity of RDS.

**Conclusion:** The decrease in serum albumin within the first day after birth was closely related to the occurrence of RDS in late-preterm infants.

## Introduction

Respiratory distress syndrome (RDS) is one of the most common diseases and a significant cause of mortality and morbidity in newborns. Common clinical manifestations of RDS include progressive dyspnea shortly after birth, cyanosis, and respiratory failure. Severe RDS is life-threatening and needs timely respiratory support as well as other treatment. Prematurity has long been recognized as the leading cause of RDS. It mainly occurs in premature infants <34 weeks of age with immature lung structure and insufficient production of pulmonary surfactant. However, recent studies have shown that the incidence of RDS in late preterm infants was also significantly higher than in term infants, and RDS at 34–36 weeks was associated with increased morbidity (1, 2).

Serum albumin has a variety of physiological functions, including protein binding and transport, colloid osmotic pressure maintenance, free radical scavenging, and changing vascular permeability alteration. A variety of pathophysiological processes can lead to a decrease in serum albumin and, finally, poor prognosis (3). Hypoalbuminemia has been shown to be a valid predictor of mortality in adult patients, but the prognostic value in neonates is not fully understood. Few studies have suggested that serum albumin level on the first day of life can also be used as an indicator of the prognosis in preterm infants (4, 5). We hypothesized that alveolar-capillary permeability increased during RDS, leading to an increase of protein leakage into the alveolar space, finally resulting in a decrease of serum albumin. Therefore, we aimed to explore the relationship between maternal-neonatal serum albumin levels and RDS in late preterm infants.

## Materials and Methods

### Study Population

This was a retrospective study of 56 late-preterm newborns (34 weeks ≤ gestational age <37 weeks) diagnosed with RDS admitted to the neonatal intensive care unit (NICU) of the Obstetrics and Gynecology Hospital of Fudan University between January 2018 to July 2019. Another 56 late-preterm infants without respiratory symptoms born in the same period were collected as the control group. These infants were admitted mainly because of low birth weight. Diagnostic criteria for RDS include (1) acute onset, progressive dyspnea shortly after birth. Present with clinical manifestation such as tachypnea, cyanosis, positive three-concave sign, or grunting. Generally, these infants need non-invasive ventilation or mechanical ventilation; (2) typical chest X-ray findings, such as fine granular densities, air bronchogram sign, ground-glass opacity, or white lungs. The exclusion criteria were as follows: meconium aspiration syndrome, pneumothorax, congenital heart disease, respiratory system abnormalities or central nervous system abnormalities, genetic metabolic disease, multiple pregnancies, sepsis, perinatal asphyxia, and death. Differential diagnosis of RDS mainly includes: 1.transient tachypnea of the newborn (TTN): TTN developed dyspnea a few hours after birth, but the course of TTN was short and the clinical manifestations are relatively mild, The chest X-ray findings are mainly alveolar, interstitial and interlobular pleural effusion. 2. congenital pneumonia: Present with clinical manifestation such as tachypnea and grunting without a progressive course. The chest X-ray findings are usually uneven distribution of exudation. Hypoalbuminemia was defined as serum albumin <30 g/L.

### Data Collection

Blood samples of pregnant women were routinely collected after hospitalization for labor or before cesarean section, and blood samples of newborns were routinely collected within 24 h after admission to NICU. Liver function was measured using venous blood samples, and the serum albumin level (g/L) was recorded. Other clinical data of mother and infant were collected, including gestational age, birth weight, gender, small for gestational age (SGA)/large for gestational age (LGA), *in vitro* fertilization (IVF), maternal age, parity, hyperlipidemia, pregnancy hypertension (pre-eclampsia, chronic hypertension), gestational diabetes mellitus, placental abruption, placenta previa, anemia, Group B streptococcus infection, antenatal corticosteroid therapy, premature rupture of membrane, uterine rupture, intrauterine distress, mode of delivery (vaginal birth or cesarean section), amniotic fluid pollution, and umbilical cord around fetal neck.

All included infants with RDS were followed up to assess disease progression. Chest X-ray finding, surfactant administration and the need for mechanical ventilation were recorded for evaluating the severity of RDS. RDS grading based on chest X-ray findings: grade I—fine, diffuse reticulogranular pattern over the lung fields; grade II—a denser lung with the presense of air bronchogram; grade III—increased density and the presence of air bronchograms beyond the heart border; grade IV—white lung.

### Statistical Analysis

Statistics were analyzed using IBM SPSS Statistics version 24.0 and MedCalc Statistical Software version 14.8.1. Measurement data were expressed as $\bar{x}\pm s$, which was compared between the two groups using an independent sample *t*-test or analysis of variance. Enumeration data were compared between the two groups using the Chi-square test or the Fisher exact test. Variables with statistical significance in univariate analysis and variables that clinically considered to effect RDS were included in the binary logistic regression analysis. Receiver Operating Characteristic (ROC) curve analysis was performed to determine the sensitivity and specificity of cutoff albumin level used to predict RDS in late-preterm infants. *P* < 0.05 was considered statistically significant.

## Results

This study included 56 late-preterm infants with RDS and 56 late-preterm infants without respiratory symptoms in the same period. Newborns in the RDS group were born at a more immature gestational age than neonates in the control group (34.92 ± 0.69 vs. 35.55 ± 0.83, *P* < 0.01). There was no statistical difference between the two groups in terms of birth weight, gender, LGA/SGA, and IVF (*P* > 0.05) (shown in Table 1). The percentage of primipara in the RDS group was lower than in the control group, and the proportion of placenta previa was higher than in the control group (*P* < 0.01). There was no statistical difference in maternal age and other complications during pregnancy (including hypertension and diabetes) between infants with and without RDS (*P* > 0.05) (shown in Table 2). Infants in the control group were more often exposed to antenatal corticosteroid therapy and to have been delivered by vaginal delivery (*P* < 0.01). There were no differences between the two groups with respect to premature rupture of membrane, intrauterine distress, amniotic fluid pollution, and umbilical cord around fetal neck (shown in Table 3).

**Table 1:** Baseline characteristics of 112 preterm infants.

**Characteristics**	**RDS group**	**Non-RDS group**	***P*-value**
	**(*n* = 56)**	**(*n* = 56)**	
Gestational age, weeks	34.92 ± 0.69	35.55 ± 0.83	0.000
Birth weight, g	2,493.39 ± 382.53	2,473.57 ± 413.64	0.793
Gender, male, *n* (%)	38 (67.86)	30 (53.57)	0.122
Small for gestational age, *n* (%)	1 (1.79)	4 (7.14)	0.360
Large for gestational age, *n* (%)	2 (3.57)	3 (5.36)	1.000
*In vitro* fertilization, *n* (%)	4 (7.14)	4 (7.14)	1.000

*RDS, respiratory distress syndrome. Values are presented as n (%) or means ± SD*.

**Table 2:** Baseline characteristics and pregnant complication of 112 pregnant women.

**Characteristics**	**RDS group**	**Non-RDS group**	***P*-value**
	**(*n* = 56)**	**(*n* = 56)**	
Age, years	31.75 ± 3.87	31.21 ± 4.73	0.513
Primipara, *n* (%)	34 (60.71)	47 (83.93)	0.006
Hyperlipemia, *n* (%)	2 (3.57)	3 (5.36)	1.000
Hypertension, *n* (%)	13 (23.21)	18 (32.14)	0.291
GDM, *n* (%)	14 (25.0)	8 (14.29)	0.154
Placental abruption, *n* (%)	4 (7.14)	2 (3.57)	0.675
Placenta previa, *n* (%)	12 (21.43)	2 (3.57)	0.004
Anemia, *n* (%)	8 (14.29)	4 (7.14)	0.222
GBS infection, *n* (%)	3 (5.36)	5 (8.93)	0.714

*RDS, respiratory distress syndrome; GDM, gestational diabetes mellitus; GBS, group B Streptococcus. Values are presented as n (%) or means ± SD*.

**Table 3:** Perinatal characteristics of 112 preterm infants.

**Characteristics**	**RDS group**	**Non-RDS group**	***P*-value**
	**(*n* = 56)**	**(*n* = 56)**	
Antenatal steroids, *n* (%)	22 (39.29)	36 (64.29)	0.008
PROM, *n* (%)	17 (30.36)	27 (48.21)	0.053
Fetal distress, *n* (%)	11 (19.64)	11 (19.64)	1.000
Vaginal delivery, *n* (%)	12 (21.43)	27 (48.21)	0.003
Amniotic fluid pollution, *n* (%)	3 (5.36)	3 (5.36)	1.000
Umbilical cord around fetal neck, *n* (%)	6 (10.71)	6 (10.71)	1.000

*RDS, respiratory distress syndrome; PROM, premature rupture of membrane. Values are presented as n (%)*.

The mean serum albumin of 112 pregnant women was 33.49 ± 3.30 g/L, and the mean serum albumin of 112 newborns was 34.18 ± 3.25 g/L. Pearson linear correlation analysis showed that there was no statistically significant correlation in serum albumin levels between pregnant women and newborns (*P* > 0.05). The mean serum albumin level of pregnant women in the RDS group was 33.38 ± 3.31 g/L, and 6 cases (10.71%) had hypoalbuminemia. The mean serum albumin level of pregnant women in the control group was 33.60 ± 3.31 g/L, and 4 cases (7.14%) had hypoalbuminemia, there was no significant difference between the two groups. However, serum albumin level in newborns with RDS was significantly lower than that in the control group (32.70 ± 2.48 vs. 35.66 ± 3.27 g/L, *P* < 0.01). The RDS group had more cases of hypoalbuminemia (3 cases, 5.36%) than the control group (0 cases), but the difference did not reach statistical significance (shown in Table 4).

**Table 4:** Maternal-neonatal serum albumin level of the two group.

**Characteristics**	**RDS group**	**Non-RDS group**	***P*-value**
	**(*n* = 56)**	**(*n* = 56)**	
Serum albumin level of pregnant women, g/L	33.38 ± 3.31	33.60 ± 3.31	0.711
Hypoalbuminemia in pregnant women, *n* (%)	6 (10.71)	4 (7.14)	0.508
Serum albumin level of infant, g/L	32.70 ± 2.48	35.66 ± 3.27	0.000
Hypoalbuminemia in infant, *n* (%)	3 (5.36)	0 (0.0)	0.079

*RDS, respiratory distress syndrome. Values are presented as n (%) or means ± SD*.

Potential confounding factors, including maternal albumin level, neonatal albumin level, hypoalbuminemia in newborns, gestational age, gender, primipara, hypertension, diabetes, placenta previa, antenatal corticosteroid therapy, pre-mature rupture of membrane and delivery mode, were analyzed using logistic regression analysis. After adjustment for gestational age, the results suggested that neonatal serum albumin levels, placenta previa, and delivery mode were independent influencing factors of RDS in late-term preterm infants (shown in Table 5). In RDS group, 41 infants had chest X-ray graded II, and 15 infants had chest X-ray graded III or IV. In terms of surfactant administration, 1 infant did not use surfactant, 51 infants used once and 4 infants used twice. Thirty-nine infants used non-invasive ventilation for respiratory support while 17 infants required invasive ventilation. However, albumin level revealed no correlation with grade of RDS, times of surfactant use and type of ventilation (shown in Table 6).

**Table 5:** Logistic analysis of multi-factor for late-preterm neonatal RDS.

**Risk factors**	**OR**	**95% CI**	***P*-value**
Serum albumin level of infant	0.721	0.574, 0.905	0.005
Placenta previa	83.213	8.725, 793.663	0.000
Vaginal delivery	0.014	0.002, 0.094	0.000

**Table 6:** Levels of albumin in relation to neonatal clinical characteristics among the RDS group (*n* = 56).

**Variables**	**Number (%)**	**Albumin (g/L)**	***P*-value**
**RDS radiological grade**			
Grade II	41 (73.21)	32.63 ± 2.47	0.759
Grade III and IV	15 (26.79)	32.87 ± 2.58	
**Surfactant**			
None	1 (1.79)	33.00 ± 0	0.795
Once	51 (91.07)	32.63 ± 2.50	
Twice	4 (7.14)	33.50 ± 2.65	
**Mechanical ventilation**			
Invasive (CMV, HFV)	17 (30.36)	32.35 ± 2.62	0.499
Non-invasive (CPAP, NIPPV)	39 (69.64)	32.84 ± 2.43	

*Values are presented as n (%) or means ± SD*.

**Figure 1:**
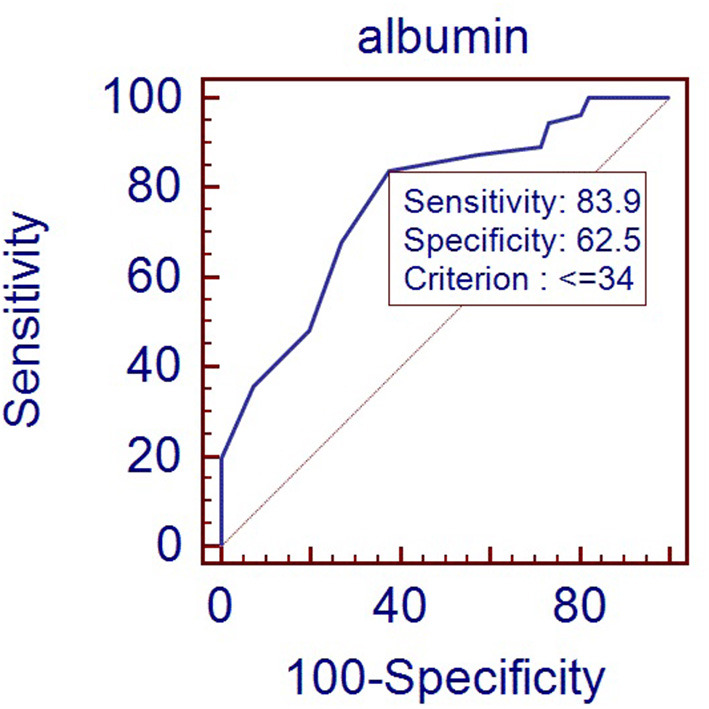
ROC curve for albumin level predicting RDS in late-preterm infants (Area under curve: 0.77, *p* < 0.001).

Figure 1 showed the ROC curve using albumin level to predict RDS in late-preterm infants. The area under the curve (AUC) was 0.77. Using the albumin cutoff level of 34 g/L provides a sensitivity of 83.9% with a specificity of 62.5%.

## Discussion

In this study, we evaluate the relationship between maternal-neonatal serum albumin levels and RDS in late-preterm infants. The main findings are that there is no significant correlation between serum albumin levels in pregnant women and infants. Serum albumin level in infants with RDS is significantly lower than infants without RDS within the first day after birth. Other risk factors associated with RDS include placenta previa and delivery mode.

Because albumin cannot be transported through the placenta, all albumin in the fetus is synthesized by the fetus itself. Amino acids are largely transported to the fetus through active transport to provide energy and materials for protein synthesis. Our study finds that although compared with the control group, albumin level of pregnant women in the RDS group shows the same downward trend as that of infants, there is no correlation between albumin levels of mothers and infants. Van der Akker et al. (6) proposed that after adjustment for weight, premature fetus had the highest albumin synthesis rate, the term fetus was the second, and the pregnant women had the lowest synthesis rate. This finding supports our conclusion. It has been reported that the albumin level at birth is associated with gestational age and postnatal age. Larger gestational age and heavier birth weight ensure higher albumin levels. Besides, antenatal corticosteroid therapy may activate the liver's ability to synthesize and thereby increase albumin levels in newborns (3, 5). This is consistent with our study; the proportion of antenatal corticosteroid therapy and albumin level in the control group were both higher than those in the RDS group.

Albumin level has been identified in adults as an evaluation index of acute respiratory distress syndrome, but there have been few studies in neonates. Bland et al. (7) studied 2200 infants and found that 33 of the 34 infants with idiopathic RDS had a cord-blood total protein concentration of 4.6 g per 100 ml or less. They proposed that the large or mature infant with low cord proteins and respiratory distress should be viewed as a likely victim of RDS. Moison et al. (8) confirmed that compared to well-preterm infants, those with RDS had a lower albumin level. Jang et al. (9) retrospectively analyzed the nutritional markers of 94 newborns admitted to the NICU at birth. They observed that the need for respiratory support on the first day of life decreased 0.001-fold for every 1 g/dL increase in albumin (95%CI, 0.000–0.136, *P* = 0.009) levels and finally proposed that nutritional status at birth was related to the need for respiratory support on the first day after birth. Hyun et al. (10) reviewed medical records of 564 extremely low birth weight infants. They proposed that lowest serum albumin level was the most effective predictor for mortality in these infants >7th postnatal days. All these studies suggest that serum albumin level may be used as a prognostic indicator of mortality in premature infants, especially respiratory diseases.

In our study, albumin level of newborns in the RDS group is significantly lower than that in the control group, and the proportion of hypoalbuminemia is higher in the RDS group (but the difference was not statistically significant). Studies (4, 7, 8, 11) have suggested that increased capillary permeability during RDS causes albumin to escape into the alveolar space. In addition, during disease states, inflammatory mediators can directly inhibit the transcription of genes responsible for albumin synthesis, limiting albumin synthesis. And in response to the prioritization of protein synthesis, the synthesis of acute response protein is up-regulated. Therefore, the albumin level decreases. However, we found no relationship between albumin level and the severity of RDS. This may be because the severity of RDS in late-preterm infants is usually lower than that in premature infants <34 weeks of age (12).

Our results suggest that using the albumin cutoff level of 34 g/L provides a sensitivity of 83.9% with a specificity of 62.5%. The reference range of albumin for newborns in our country is 32–48 g/L (13). So even if the albumin level is within the lower limit of the normal range, for late-preterm infants with respiratory distress, we need to be alert to the possibility of RDS. Several other studies also discussed the albumin's prospective value in infants. Torer et al. (4) enrolled 199 preterm infants and studied serum albumin level on the first day after birth. They found that albumin level <25 percentile was an independent predictor of mortality. Albumin concentration lower than 27.2 g/L was associated with mortality, with a sensitivity of 71% and a specificity of 86%. Jon F. Watchko et al. (5) studied 382,190 paired albumin and bilirubin levels across 164,401 neonates. They found that neonates with serum albumin levels <2.5 g/dL was at higher risk of death compared with infants who did not exceed these levels.

Besides, our research shows that placenta previa and cesarean section are also risk factors for RDS in late preterm infants. Many studies have confirmed similar conclusions. Lin et al. (14) found that preterm infants born to mothers with placenta previa had a higher risk for RDS that controls. But only in infants treated with antenatal steroid it played an independent role. Tsuda et al. (15) also proposed that placenta previa in itself is a risk factor for transient tachypnea of the newborn. Ahn et al. (16) stated that anterior placenta previa detected during the second trimester, irrespective of whether the placenta will migrate in the third trimester, may be an independent risk factor for neonatal RDS. Berthelot-Ricou et al. (17) retrospectively studied near term infants in 5 years. They found that late preterm infants born via elective cesarean section are at high risk for RDS. This was also supported by many other researches (12, 18, 19).

There are some limitations in our research. First, our research focused on late-preterm infants and the incidence of RDS in late-preterm infants is relatively low, so the sample size of the study is small and needs to be further expanded to enhance statistical significance. Second, we only studied the albumin level on the first day instead of the lowest albumin level or the change trend of albumin level. However, it is difficult to predict the appearance time of the lowest albumin level during the course of the disease, so the comparison of the lowest albumin level is not very operative. Using albumin level of the first day is more meaningful in clinical practice. Follow-up studies can include the change trend of albumin in the first 3 days as an indicator.

In summary, our study extends the knowledge into serum albumin's prospective value in newborns. We find that the maternal albumin level is not related to the infant albumin level. More importantly, a decrease in serum albumin level on the first day after birth is an independent risk factor for RDS in late preterm infants. Low albumin levels suggest that more active respiratory support measures may be needed.

## Data Availability Statement

The raw data supporting the conclusions of this article will be made available by the authors, without undue reservation.

## Ethics Statement

Ethical review and approval was not required for the study on human participants in accordance with the local legislation and institutional requirements. Written informed consent to participate in this study was provided by the participants' legal guardian/next of kin.

## Author Contributions

QY and X-qY conceptualized and designed the study. QY, X-qY, and FL collected the data. X-qY analyzed the data. QY and J-mW drafted the initial manuscript. All authors contributed to the interpretation of the data and contributed to the final draft of the manuscript.

## Conflict of Interest

The authors declare that the research was conducted in the absence of any commercial or financial relationships that could be construed as a potential conflict of interest.

## Publisher's Note

All claims expressed in this article are solely those of the authors and do not necessarily represent those of their affiliated organizations, or those of the publisher, the editors and the reviewers. Any product that may be evaluated in this article, or claim that may be made by its manufacturer, is not guaranteed or endorsed by the publisher.
